# Supplementary material for: Vitamin D Status among Women in a Rural District of Nepal: Determinants and Association with Metabolic Profile—A Population-Based Study
Source: Nutrients. 2022 May 31;14(11):2309. doi: 10.3390/nu14112309 (PMC9182746; doi:10.3390/nu14112309)
Supplement: Supplementary file 1 [file nutrients-14-02309-s001.zip › nutrients-1728440-supplementary.pdf]

Table S1. Department of Clinical Biochemistry, Dhulikhel Hospital.

Test	Unit	Reference values	Method
Glycosylated Hb/HbA1c	mmol/mol	Normal: 26-38 mmol/mol<br>Pre-Diabetes:39-47 mmol/mol<br>Diabetes: ≥ 48 mmol/mol	HPLC
25-OH-Vit D Total	nmol/L	Vitamin D sufficiency: >75 nmol/L<br>Vitamin D insufficiency: 25-75 nmol/L<br>Vitamin D deficiency: < 25 nmol/L	LIAISON (CLIA)
Calcium-Total	mmol/L	2.1-2.6 mmol/L	C
Phosphorus, Inorganic	mmol/L	0.8-1.5 mmol/L	C
Cholesterol, Total	mmol/L	<5.2 mmol/L: Desirable; 5.2-6.2 mmo/L: Borderline high; High> 6.2 mmol/L	CHODA
HDL-Cholesterol	mmol/L	Desirable M 1.6 mmol/L & F> 1.9 mmol/L, acceptable: 0.9 -1.2 mmol/L Low: 0.9 mmol/L	Direct estimation
LDL-Cholesterol	mmol/L	< 2.6 mmol/L: Optimal, 2.6-3.3 mmoL/L; Above, 3.3-4.1 mmol/L; Borderline High, 4.1-4.9 mmol/L; High, > 4.9 mmol/L; Very High	Direct estimation
Triglycerides	mmol/L	< 1.7 mmol/L; Normal, 1.7- 2.2 mmol/L; Borderline High, 2.3- 5.6 mmol/L; High, > 5.6 mmol/L; very High	Enzyme
PTH	pg/mL	6.5 – 36.8 pg/mL	LIASION

HPLC; High Performance Liquid Chromatography

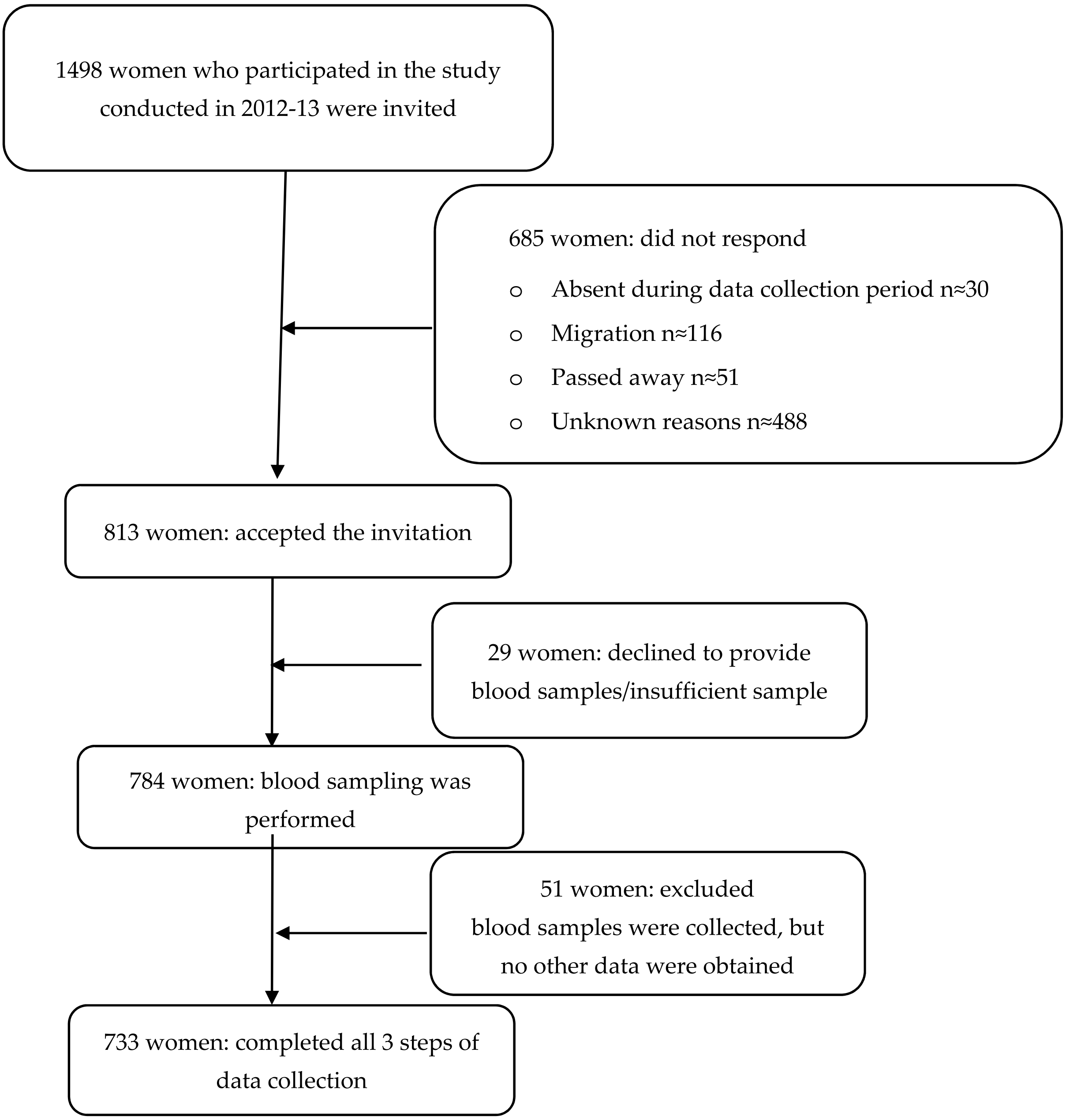

Figure S1: Flow diagram of study population.
